# Advances in the regulation of bone metabolism by central nuclei: a new perspective on the brain-bone axis

**DOI:** 10.3389/fnins.2026.1801524

**Published:** 2026-04-10

**Authors:** Guanhao Yang, Xuan Huang, Duorun Qiu, Anzhao Wang, Denghui Liu, Zhongtang Liu

**Affiliations:** 1 Department of Orthopedics, The First Affiliated Hospital of Naval Medical University, Shanghai, China; 2 Department of Anesthesiology, The Second Affiliated Hospital of Naval Medical University, Shanghai, China; 3 Department of Orthopedics, The 906th Hospital of the Joint Logistics Support Force of PLA, Ningbo, Zhejiang, China

**Keywords:** bone metabolism, brain-bone axis, central nervous system, central nuclei, osteoporosis

## Abstract

The convergence of neuroscience and bone biology has led to the profound conceptualization of the “brain-bone axis,” revealing the intricate connection between the central nervous system and bone metabolism. Central nuclei, defined as structures composed of neurons with similar functions, play a pivotal role in the regulation of bone metabolism. Research has demonstrated that central nuclei can modulate bone metabolism either directly via nerve fiber projections or indirectly through neuroendocrine pathways. Despite significant progress, the precise mechanisms by which central nuclei regulate bone metabolism and the collaborative communication networks among these nuclei remain poorly understood. This review systematically summarizes recent advances in central nuclei across the entire brain that regulate bone metabolism and their complex mechanisms. Moreover, it discusses current challenges and potential future research directions in the field. The ultimate aim is to provide novel insights for developing neuromodulatory therapeutic strategies against bone metabolic disorders.

## 1 Introduction

Bone metabolism is a dynamic equilibrium process through which the skeleton achieves self-renewal and maintains homeostasis. This process is fundamentally characterized by the tight coupling between osteoclast-mediated bone resorption and osteoblast-mediated bone formation (Bao et al., 2020; Srivastava et al., 2022). Under physiological conditions, bone resorption and bone formation remain balanced. However, disruption of this equilibrium leads to abnormalities in skeletal structure or function, resulting in disorders such as osteoporosis or osteopetrosis, thereby compromising bone integrity and health (Chen et al., 2018). Consequently, elucidating the regulatory mechanisms of bone metabolism is crucial for understanding the pathogenesis of related diseases. Traditional research has primarily focused on local regulatory mechanisms of bone metabolism, including the modulation of calcium and phosphate metabolism by parathyroid hormone (PTH), the promotion of intestinal calcium absorption by vitamin D, and the suppression of bone resorption by estrogen (Elahmer et al., 2024). Nevertheless, this perspective falls short in explaining bone metabolic disorders associated with comorbid conditions, such as bone loss induced by Alzheimer’s disease (AD) (Pu et al., 2020), chronic stress (Stefanaki et al., 2023), and circadian rhythm disruption (Swanson et al., 2018). These conditions suggest that skeletal homeostasis may be governed by systematic regulation, while the role of the central nervous system (CNS) has long been overlooked (Kelly et al., 2020; Massaccesi et al., 2025; Otto et al., 2020).

Serving as the core of the CNS regulating bone metabolism, central nuclei are structures formed by clusters of neurons with similar functions (Hatanaka et al., 2016). In the early 2000s, groundbreaking research by Gérard Karsenty’s team definitively identified specific nuclei involved in bone metabolism and their key roles: the ventromedial hypothalamus (VMH) and arcuate nucleus (ARC) were shown to remotely regulate bone mass in mice through the leptin pathway, independently of leptin’s classical role in energy metabolism (Takeda et al., 2002). This seminal discovery gave rise to the revolutionary concept of the “brain-bone axis (BBA)”, which fundamentally represents bidirectional communication between central nuclei and the skeletal system (Shi and Chen, 2024; Dimitri and Rosen, 2017; Martiniakova et al., 2025). Within this axis, central nuclei act as supreme command centers, which are interconnected through intricate neural circuits to integrate internal and external signals and generate regulatory commands (Sporns et al., 2005). Specifically, ARC serves as a pivotal gateway for sensing peripheral metabolic signals, modulating the balance between energy metabolism and bone metabolism (Na et al., 2022; Chen et al., 2024; Idelevich et al., 2020). The suprachiasmatic nucleus (SCN), as the master of circadian clock, maintains bone homeostasis by synchronizing circadian rhythms (Lucassen et al., 2016). The lateral hypothalamus (LH), a critical brain region governing appetite, modulates bone homeostasis through the secretion of orexin (Wei et al., 2014). These outputs are mediated by the peripheral nervous system (e.g., autonomic and sensory nerves) and rely on neurotransmitters and hormones (such as norepinephrine (NE) and leptin) for signal transmission to the bone (Liang et al., 2025). Specifically, the sympathetic nervous system (SNS) regulates bone metabolism mainly via NE, while the parasympathetic nervous system (PSNS) counteracts SNS activity through acetylcholine (ACh). Sensory nerves secrete neuropeptides like calcitonin gene-related peptide (CGRP) and substance P (SP) to promote osteogenesis and angiogenesis. Additionally, neurotrophins such as nerve growth factor (NGF) and brain-derived neurotrophic factor (BDNF) participate in neural-osseous crosstalk, synergistically maintaining bone homeostasis with central signals (Li J. et al., 2024).

**Figure 1:**
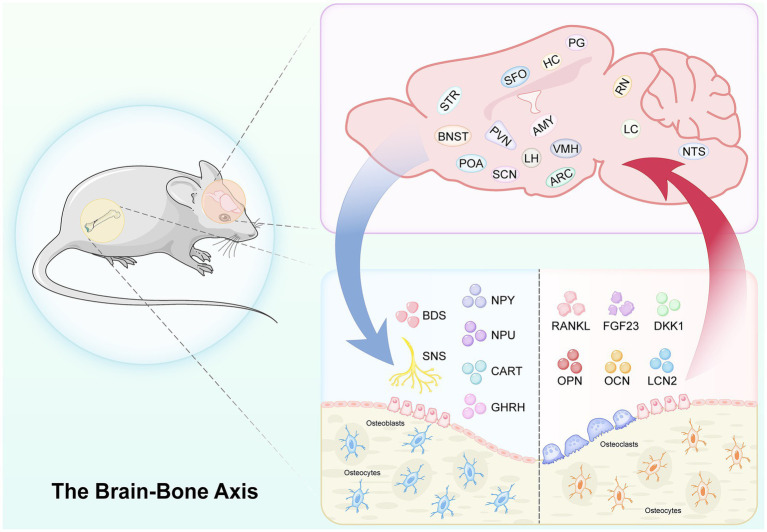
The “brain-bone axis (BBA)” represents a bidirectional communication network connecting the brain and the skeleton. Within this system, central nuclei, widely distributed across brain regions such as the diencephalon, brainstem, limbic system, and basal ganglia, function as central hubs. These nuclei not only send remote instructions to regulate bone metabolism but also respond to feedback from bone-derived factors secreted by the skeleton, thereby forming a complete regulatory circuit.

Concurrently, acting as an endocrine organ, the skeleton secretes bone-derived factors—such as osteocalcin (OCN), fibroblast growth factor 23 (FGF23), and lipocalin 2 (LCN2)—precisely target central nuclei, establishing a closed-loop regulatory network (He et al., 2025; Zheng et al., 2023). Specifically, OCN exerts multiple regulatory effects on cognitive function and the homeostasis of the CNS. OCN binds to G-protein-coupled receptor 158 (GPR158) and activates a primary cilia-dependent signaling pathway to modulate neuronal autophagy, a process that is critical for the maintenance of cognitive resilience (Rivagorda et al., 2025). In oligodendrocytes, OCN inhibits cellular differentiation and CNS myelination via G-protein-coupled receptor 37 (GPR37), thereby orchestrating the homeostasis of the myelin sheath (Qian et al., 2021). Clinical studies have further demonstrated that OCN levels in AD patients are elevated, which is negatively correlated with cerebral *β*-amyloid deposition, implying a potential association between OCN and the pathological progression of AD (Bu et al., 2025). Furthermore, LCN2 secreted by osteoblasts can translocate to the hypothalamus and bind to the melanocortin 4 receptor (MC4R), thus activating anorexigenic neural pathways (Mosialou et al., 2017). FGF23, a key mediator of calcium and phosphate metabolism secreted by osteocytes, leads to hippocampus (HC)-dependent cognitive impairment when functionally deficient (Laszczyk et al., 2019) (Figure 1).

During the above process, central nuclei serve as the core of the BBA, playing a pivotal role in bidirectional communication between the brain and bone. However, current research on central nuclei exhibits a pronounced imbalance. Extensive research has concentrated on the hypothalamic nuclei—especially their functions in metabolic signal sensing, sympathetic nerve activation, and hypothalamic–pituitary-gonadal (HPG) axis regulation (Idelevich and Baron, 2018; Chamouni et al., 2015). This narrow perspective has failed to adequately illuminate the significant role of other central nuclei throughout the brain in regulating bone metabolism. For example, the locus coeruleus (LC), being the primary source of central NE, regulates bone remodeling processes by enhancing sympathetic tone (Issidorides et al., 2004; Kajimura et al., 2014). Furthermore, limbic nuclei such as the amygdala (AMY) act as integration hubs for emotional and stress responses, significantly impacting bone metabolism under conditions of anxiety or chronic stress (Wood et al., 2016; Pahk et al., 2021). In reality, the brain processes and integrates peripheral signals via widely distributed nuclei, fine-tuning bone homeostasis through specialized neural circuits under diverse physiological and pathological conditions. Besides, the regulation of bone metabolism by central nuclei does not issue commands arbitrarily. It takes peripheral signals reflecting the physiological status of the organism as the core input, which are integrated to generate precise neural regulatory outputs. Leptin secreted by adipocytes represents a classic metabolic signal. Upon sensing this signal, nuclei including the VMH and ARC can inhibit bone formation by activating the SNS (Takeda et al., 2002), or suppress bone resorption by upregulating the expression of cocaine- and amphetamine-regulated transcript (Elefteriou et al., 2005). SNS activation serves as the hallmark of stress signals. Evidence has shown that the metabolic activity of the AMY is abnormally altered in postmenopausal women (PMW) complicated with osteoporosis (Pahk et al., 2021); in addition, the neural circuit consisting of the bed nucleus of the stria terminalis (BNST), dorsomedial VMH (VMHdm) and nucleus tractus solitarius (NTS) can further enhance SNS activity, thereby inducing bone loss (Yang F. et al., 2020). Temporal signals are modulated through circadian rhythms. As the master pacemaker driving the behavioral rhythms (Mohawk et al., 2012), the SCN maintains bone homeostasis by synchronizing circadian clock rhythms, while circadian rhythm disruption directly impairs skeletal health (Arafa and Emara, 2020).

This review aims to transcend the “hypothalamo-centric” perspective by synthesizing evidence on central nuclei across the entire brain that regulate bone metabolism. Integrating cross-species model systems and clinical research data, we systematically delineate the anatomical localization and functional contributions of nuclei within the diencephalon, brainstem, limbic system, and basal ganglia. Our study leverages diverse methodologies including optogenetic or chemogenetic approaches (Zhao et al., 2024), neural circuit tracing (Liu et al., 2022), and gene editing technologies (Swiech et al., 2015). This review constructs a functional atlas of integrated circuits across multiple nuclei, advancing transformative insights into neural mechanisms of bone metabolic disorders.

Having introduced the overarching concept of the BBA and the various central nuclei involved, we now analyze these nuclei in detail, beginning with those located in the diencephalon, which have been the most extensively studied.

## 2 Diencephalon

### 2.1 Hypothalamus

#### 2.1.1 Arcuate nucleus (ARC)

Positioned at the hypothalamic base adjoining the third ventricle and median eminence, the ARC features a selectively permeable blood–brain barrier (BBB) (Li et al., 2019). This anatomical architecture positions the ARC as a pivotal hub for direct sensing of peripheral metabolic signals (e.g., leptin, insulin) (Na et al., 2022). Within the ARC, orexigenic agouti-related peptide/neuropeptide Y (AgRP/NPY) neurons and anorexigenic pro-opiomelanocortin/cocaine -and amphetamine-regulated transcript (POMC/CART) neurons form functionally antagonistic populations that coordinate systemic energy homeostasis (Chen et al., 2024; Idelevich et al., 2020).

##### 2.1.1.1 AgRP/NPY neurons bidirectionally regulate bone metabolism

AgRP neurons autonomously sense energetic status and predominantly promote bone formation. Pyruvate dehydrogenase kinase 1 (PDK1) serves as a central signaling node for leptin and insulin in the ARC, governing feeding behavior and energy homeostasis (Zhang et al., 2018). PDK1 enhances bone formation through the growth hormone-releasing hormone–growth hormone–insulin-like growth factor 1 (GHRH-GH-IGF1) axis (Sasanuma et al., 2017). Uncoupling protein 2 (UCP2), a master regulator of cellular bioenergetics (Monteiro et al., 2021), is highly expressed in AgRP neurons (Andrews et al., 2008). Systemic *Ucp2* knockout (*UCP2^−/−^*) induces significant bone loss, whereas AgRP neuron-specific UCP2 restoration rescues this low-bone-mass phenotype (Kim et al., 2015). Sirtuin 1 (Sirt1) is an NAD^+^-dependent deacetylase that regulates negative energy balance (Dietrich et al., 2010). Previous studies indicate its role in promoting chondrocyte proliferation and differentiation (Li et al., 2016). AgRP neuron-specific *Sirt1* knockout mice (*AgRP^Sirt1−/−^*) exhibit a significant reduction in bone mineral density (BMD) (Kim et al., 2015). Paradoxically, distinct interventions targeting AgRP neurons yield opposing phenotypes: diphtheria toxin-mediated ablation of AgRP neurons reduces bone mass (Kim et al., 2015), whereas AgRP deletion increases cortical and trabecular bone mass in mice, with male-specific predominance (Enriquez et al., 2021). The core mechanism lies in the difference in signal output integrity. AgRP neurons release AgRP, NPY, and GABA to regulate bone metabolism via multiple pathways, and the ablation of these neurons results in the elimination of all three signals. In contrast, AgRP gene deletion only removes the AgRP peptide, while preserving NPY/GABA secretion and neuronal circuit function (Wu et al., 2009).

In contrast to the complex role of AgRP neurons, NPY neurons act as negative regulators of bone homeostasis. *NPY*-deficient mice (*NPY^−/−^*) exhibit significantly increased bone mass, whereas ARC-specific *NPY* overexpression via recombinant adeno-associated virus (rAAV) induces osteopenia (Meunier et al., 2009). This bone loss is attributed specifically to NPY within the ARC, as hippocampal *NPY* overexpression fails to alter bone metabolism (Baldock et al., 2005). In addition, NPY regulates the balance between bone metabolism and fat metabolism. Skeleton interoceptive signaling ascends to the ARC, reducing NPY production and thereby inducing adipose tissue lipolysis. The free fatty acids (FFAs) provide energy substrates for osteoblasts, ultimately promoting bone formation (Gao et al., 2021).

##### 2.1.1.2 POMC/CART neurons profoundly influence bone metabolism

POMC neurons establish a central-peripheral antagonistic loop in bone homeostasis through estrogen receptor *α* (ERα) signaling. Peripherally, activation of ERα promotes osteoblast survival and inhibits osteoclast differentiation (Khalid and Krum, 2016). However, POMC neuron-specific ERα deletion (POMC-*ERα^−/−^*) increases cortical bone mass and mechanical strength in female mice. The paradoxical bone phenotype of POMC-*ERα^−/−^* mice stems from the decoupling of central and peripheral ERα pathways. Peripheral ERα maintains basal osteoprotection, while central POMC-ERα acts as a “brake” on excessive bone accrual by restraining POMC neuron activity. Deleting this central brake releases compensatory osteogenic signaling that synergizes with peripheral ERα-mediated osteoprotection, ultimately leading to enhanced bone mass and mechanical strength (Ohlsson et al., 2016). This mechanism highlights the complexity of central-peripheral crosstalk in bone metabolism and underscores the critical role of ERα signaling in the BBA.

Moreover, CART neurons co-expressed with POMC neurons suppress bone resorption. Leptin action on the ARC upregulates CART expression, which inhibits receptor activator of nuclear factor kappa B ligand (RANKL) to reduce osteoclast differentiation. In *Cart* knockout mice (*Cart^−/−^*), bone resorption is significantly increased, resulting in a low-bone-mass phenotype (Elefteriou et al., 2005).

##### 2.1.1.3 Other neurons expand the regulatory dimensions of bone metabolism

Kisspeptin (Kiss1) neurons in the ARC express high levels of ERα (Herber and Ingraham, 2019). *ERα* deletion specifically in Kiss1 neurons increases bone mass in intact and ovariectomized (OVX) female mice (Herber et al., 2019). Cellular communication network factor 3 (CCN3), secreted from Kiss1 neurons, possesses potent osteogenic activity. It is specifically upregulated during lactation when estrogen levels plummet, promoting bone formation and remodeling. CCN3 accelerates fracture healing in young and aged mice, suggesting a potential therapeutic promise for bone fracture, which requires further validation in large-sample and long-term animal models (Babey et al., 2024).

Furthermore, ablation of MSG-sensitive neurons in the ARC induces hypogonadism. However, contrasting with established knowledge that hypogonadism typically increases bone resorption and reduces bone mass (Jilka, 1998), this ablation does not cause bone loss. The underlying mechanism is inferred to involve a concurrent increase in bone resorption accompanied by a more pronounced elevation in bone formation, which offsets the bone loss induced by hypogonadism. Following estradiol treatment to correct hypogonadism, bone resorption normalizes while bone formation remains elevated, resulting in high bone mass. Nevertheless, MSG treatment does not inhibit the antiosteogenic function of leptin in leptin-deficient (*ob/ob*) mice. This confirms that MSG-sensitive neurons control bone formation in a leptin-independent manner (Elefteriou et al., 2003).

**Figure 2:**
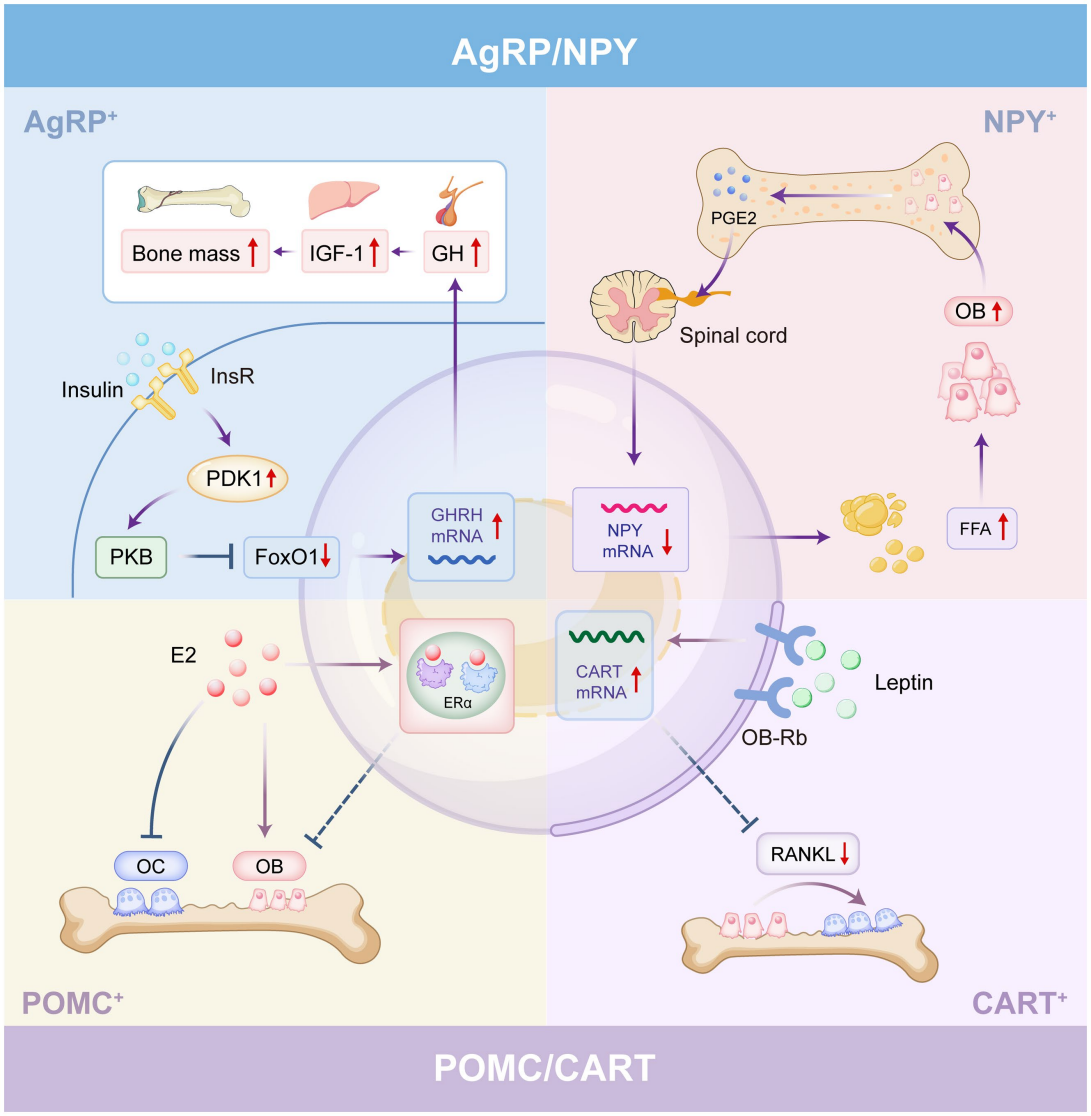
The ARC regulates bone mass through different types of neurons. In AgRP neurons, PDK1 activation suppresses FoxO1, enhancing bone formation via the GHRH–GH–IGF1 axis. Skeletal interoceptive signaling relayed to NPY neurons represses NPY gene expression and promotes adipose tissue lipolysis, thereby facilitating osteoblast differentiation. While peripheral activation of ERα promotes osteoblast proliferation and inhibits osteoclast differentiation, its activation within POMC neurons paradoxically suppresses bone mass, a process that requires further elucidation. Leptin action on the ARC upregulates CART expression, which inhibits RANKL to reduce osteoclast differentiation and maintain bone mass.

In summary, the ARC serves as a central signal integrator harboring multiple key neuronal populations that regulate bone mass. Among these, functionally antagonistic AgRP/NPY and POMC/CART neurons integrate energy metabolism signals while differentially maintaining bone homeostasis. Studies reveal that both neuronal types enhance energy expenditure and increase bone mass through antagonism of activator protein 1 (AP1) by ΔFosB, a splice isoform of the AP1 transcription factor FosB, albeit via distinct mechanisms (Idelevich et al., 2018, 2020). Furthermore, Kiss1 neurons and MSG-sensitive neurons in the ARC exert significant influences on bone metabolism through distinct mechanisms. Collectively, the ARC maintains bone homeostasis through coordinated neuronal actions (Figure 2).

#### 2.1.2 Ventromedial hypothalamus (VMH)

The VMH, a bilateral oval-shaped nucleus located dorsomedially at the hypothalamic base and superior to the median eminence (Choi et al., 2013), plays a crucial role in glucose homeostasis and energy balance (Fujikawa et al., 2019). VMH is also a critical nucleus for regulating bone metabolism. It modulates bone mass by mediating SNS activity through the reception and processing of diverse biological signals.

##### 2.1.2.1 The SNS serves as the critical pathway

Studies of leptin signaling reveal SNS regulation. Secreted by adipocytes, leptin activates steroidogenic factor 1 (SF1) neurons to stimulate SNS outflow; this signal is transmitted to β_2_-adrenergic receptors (β_2_-ARs) on osteoblasts, inhibiting bone formation (Takeda et al., 2002; Cock and Auwerx, 2003).

##### 2.1.2.2 The VMH fine-tunes SNS activity

The VMH also harbors intrinsic mechanisms to dampen sympathetic tone. VMH-derived secretin (SCT) acts on secretin receptors (SCTR) within the VMH via autocrine or paracrine mechanisms, promoting phosphorylation of cAMP response element-binding protein (CREB). This signaling cascade reduces sympathetic tone and facilitates bone accrual (Zhang et al., 2024). Furthermore, Brain-derived serotonin (BDS) binds to serotonin receptor 2C (HTR2C) on VMH neurons, similarly reducing sympathetic tone via CREB phosphorylation to increase bone mass (Yadav et al., 2009; Oury et al., 2010). This mechanism partially explains the bone loss side effect of central serotonergic drugs such as selective serotonin reuptake inhibitors (SSRIs) (Modder et al., 2010).

##### 2.1.2.3 The SNS mediates pathophysiological processes

The SNS is critical for translating environmental challenges into skeletal responses. Under chronic stress, a GABAergic circuit from the BNST to SF1 neurons in the VMHdm enhances SNS activity, leading to bone loss (Yang F. et al., 2020). Conversely, during sustained mechanical loading (e.g., orthodontic tooth movement, OTM), activation of tyrosin hydroxylase (TH)-positive neurons in the VMH accelerates osteoclast differentiation and alveolar bone remodeling via the SNS (Cao et al., 2020). VMH astrocytes further modulate this system; activation of their Gq pathway enhances SF1 neuron excitability, thereby increasing anxiety-like behavior and reducing bone mass in mice (Liu et al., 2021).

##### 2.1.2.4 An intact VMH underpins SNS regulation

The structural integrity of the VMH is indispensable. As nutrient status sensors (Han et al., 2014), primary cilia on VMH neurons require intact intraflagellar transport 88 (*IFT88*). Deletion of this key gene elevates bone mass by reducing SNS activity (Sun et al., 2021). The indispensability of the VMH is further confirmed by its chemical ablation, which abolishes both leptin’s anti-osteogenic effect and unloading-induced bone loss (Takeda et al., 2002; Hino et al., 2006).

**Figure 3:**
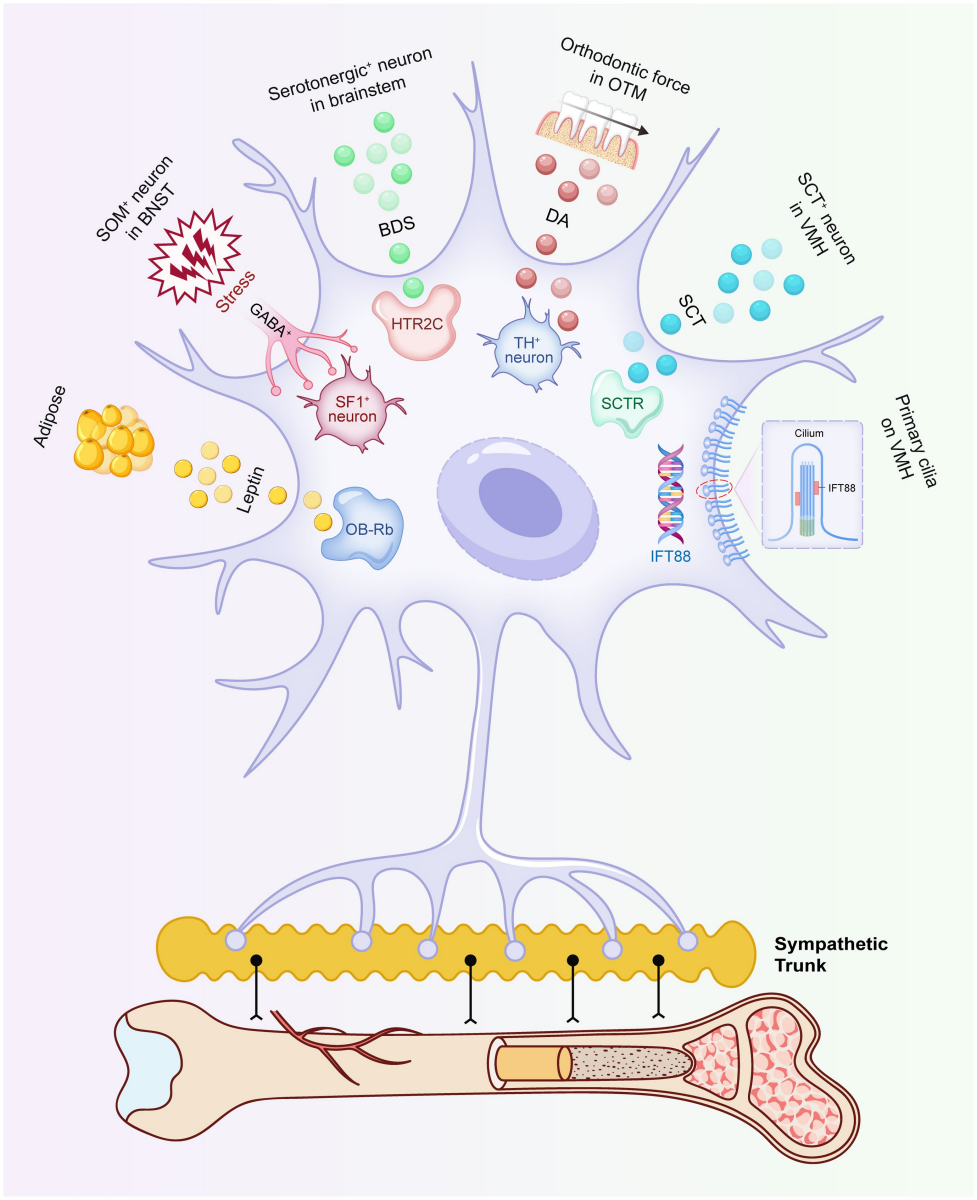
The VMH regulates bone metabolism by modulating the activity of the SNS. This process integrates a diverse array of signals and structural components, including leptin secreted by adipocytes, GABAergic inputs from SOM neurons in the BNST, BDS produced by serotonergic neurons in the brainstem, DA induced by OTM, SCT released by secretin neurons in the VMH, and primary cilia within the VMH. These signals collectively enhance or suppress SNS outflow to finely tune bone mass.

In summary, the VMH exerts multi-layered regulation on bone formation and resorption via the SNS. However, this coherent paradigm reveals unexpected complexity. Unlike in the ARC neurons, where AP1 antagonism increases both energy expenditure and bone mass, pharmacological inhibition of SF1 neurons in the VMH paradoxically dissociates these outcomes, reducing bone mass while increasing energy metabolism. This arises from sustained activation of the SNS and competitive activation of energy metabolic pathways, ultimately leading to reduced osteoblastic activity and bone loss (Idelevich et al., 2018). This phenomenon indicates a unique and refined role for the VMH in coordinating energy and bone homeostasis, warranting further investigation (Figure 3).

#### 2.1.3 Suprachiasmatic nucleus (SCN)

The SCN is bilaterally distributed in the anterior hypothalamus, dorsal to the optic chiasm and adjacent to the ventral wall of the third ventricle. Each nucleus comprises approximately 10,000 tightly coupled neurons (Patton and Hastings, 2018). The SCN serves as the central pacemaker of the mammalian circadian system (Speksnijder et al., 2024).

##### 2.1.3.1 The SCN maintains bone homeostasis by synchronizing circadian rhythms

Chronic circadian disruption compromises skeletal health. Animal studies demonstrate that 24 weeks of continuous light exposure significantly dampens rhythmicity in the SCN central pacemaker, inducing early osteoporotic microarchitectural alterations (Lucassen et al., 2016). Clinical investigations further confirm that shift workers exhibit reduced BMD and elevated fracture risk due to circadian misalignment, highlighting the essential protective role of circadian rhythms in skeleton (Quevedo and Zuniga, 2010; Feskanich et al., 2008).

##### 2.1.3.2 The SCN stabilizes circadian rhythms by coordinating the core clock gene network

As key negative regulators of the circadian clock, *period* (*Per*) genes mediate sympathetic signals that inhibit cell proliferation. Specifically, mutation of the *Per2* gene shortens the cell cycle and enhances osteoblast proliferation. Consequently, *Per2*-knockout mice exhibit a high-bone-mass phenotype. Leptin partially counteracts this inhibition by promoting cyclin D1 production, thus enhancing osteoblast proliferation and bone formation (Abe et al., 2019; Fu et al., 2005). This paradox highlights the critical role of the circadian clock gene network in modulating leptin signaling in the BBA, emphasizing that leptin’s osteoregulatory function is context-dependent and tightly linked to circadian rhythm integrity. Understanding this mechanism provides implications for treating osteoporosis associated with circadian misalignment.

##### 2.1.3.3 The SCN regulates bone metabolism via neuroendocrine and neuropeptide pathways

Via the SNS and glucocorticoids (GCs), the SCN transmits circadian signals to bone and drives diurnal oscillations in bone metabolic markers and regulatory hormones. These markers include C-terminal cross-linked telopeptide of type I collagen (CTX), procollagen type 1 N-propeptide (P1NP), and alkaline phosphatase (ALP), while the hormones involve PTH, melatonin, and ghrelin (Song et al., 2018; Fujihara et al., 2014). Additionally, specific neuropeptides within the SCN modulate bone metabolism. The neuropeptide Y6 receptor (Y6R) is specifically expressed in the SCN. *Y6R*-knockout (*Y6R*-KO) mice exhibit reduced bone mass with unaffected bone length (Yulyaningsih et al., 2014; Khor et al., 2016). This finding suggests that SCN-expressed Y6R is a potential mediator of this process, a conclusion that requires validation using SCN-specific *Y6R* knockout and activation models.

#### 2.1.4 Paraventricular nucleus (PVN)

The PVN, situated in the anterior hypothalamus near the third ventricle, consists of magnocellular neurons synthesizing oxytocin (OT) and arginine vasopressin (AVP), parvocellular neuroendocrine neurons secreting hypophysiotrophic hormones, and parvocellular pre-autonomic neurons modulating sympathetic outflow (Pyner, 2014; Qin et al., 2018). This neuronal heterogeneity forms the structural foundation for PVN-mediated regulation of bone metabolism. The PVN maintains bone homeostasis primarily through two core pathways.

##### 2.1.4.1 Neuro-endocrine pathway

Magnocellular neurons in the PVN and supraoptic nucleus (SON) co-synthesize OT and AVP, which are transported to the posterior pituitary for systemic release into circulation (Brown, 2016; Japundžić-Žigon, 2013). These hormones subsequently act on bone tissue to regulate bone mass. OT serves as a potent bone anabolic agent that directly stimulates the differentiation of osteoblasts to a mineralizing phenotype by up-regulating bone morphogenetic protein 2 (BMP2), thereby enhancing osteogenic capacity (Tamma et al., 2009). In contrast, AVP predominantly drives bone catabolic processes through suppression of osteogenic gene expression [e.g., ALP, runt-related transcription factor 2 (Runx2)] in osteoblasts. This effect is dependent on the Avp receptor 1α (Avpr1α). *Avpr1α^−/−^* mice exhibit a pronounced high-bone-mass phenotype (Sun et al., 2015).

##### 2.1.4.2 Neuro-neuronal pathway

The PVN constitutes the primary hypothalamic site governing sympathetic outflow and energy expenditure (Kvetnansky et al., 2009). The pre-autonomic neurons process afferent inputs from regions including the NTS and ARC. These integrated signals converge to modulate sympathetic activity via two principal descending routes: indirect relay through the motor pressor nucleus of the rostral ventrolateral medulla (RVLM) or direct projections to motor sympathetic preganglionic neurons (SPNs) in the thoraco-lumbal intermediolateral nucleus (IML) (Pyner, 2014; Sapru, 2013; Savic et al., 2022). This descending sympathetic tone is a critical determinant of bone mass. Concurrently, ascending feedback from bone fine-tunes this system. For instance, skeletal interoception from mechanical loading ascends to the ARC and subsequently suppresses TH expression in the PVN, which reduces sympathetic tone and consequently enhances bone formation (Gao et al., 2024).

#### 2.1.5 Lateral hypothalamus (LH)

The LH constitutes a highly heterogeneous subregion within the hypothalamus (Barbier et al., 2022). As a critical hub for appetite and arousal regulation, the LH modulates bone metabolism through secretion of orexin and melanin-concentrating hormone (MCH) (Tyree and de Lecea, 2017; Concetti and Burdakov, 2021; Arrigoni et al., 2019).

##### 2.1.5.1 Orexin regulates bone remodeling through both central and peripheral pathways

Centrally, orexin enhances bone formation via orexin receptor 2 (OX2R) in the brain, a mechanism that involves the significant reduction of circulating leptin levels. Peripherally, by binding to the orexin receptor 1 (OX1R) in bone, orexin suppresses local ghrelin levels, thereby inhibiting bone formation (Wei et al., 2014; Pizza et al., 2022). This paradoxical central-peripheral difference arises from the independence of their signaling targets: the central pathway couples energy metabolism and bone metabolism via leptin, while the peripheral pathway directly targets local bone microenvironment signals. Notably, OX1R activation initiates the Nrf2/HIF-1α pathway to reverse bone loss induced by chronic intermittent hypoxia (CIH), suggesting a therapeutic avenue for osteoporosis associated with obstructive sleep apnea (OSA) (Gu et al., 2022).

##### 2.1.5.2 MCH neurons regulate bone mass through trans-synaptic connections

MCH expressing neurons in the LH are directly connected to the bone. Chemical activation of these neurons stimulates bone marrow stromal cells (BMSCs) to drive osteogenic differentiation and bone formation. This finding closely aligns with the evidence from both humans and mice: reduced MCH neuronal activity in the LH of osteoporosis patients and decreased plasma MCH concentrations in osteoporosis mice, respectively (Prida et al., 2022; Guo et al., 2024). Thus, the MCH neuron represents a promising therapeutic target for osteoporosis.

#### 2.1.6 Preoptic area (POA)

The POA, situated at the rostral extreme of the hypothalamus, continuously monitors peripheral signals through specific circumventricular organs (CVOs): the vascular organ of the lamina terminalis (VOLT) and subfornical organ (SFO). These organs lack BBB, enabling real-time coordination of body temperature, reproductive behavior, and other physiological process (Yu et al., 2018; Tabarean et al., 2010; Tsuneoka and Funato, 2021), ultimately coupling with bone metabolism.

##### 2.1.6.1 POA maintains bone homeostasis via gonadal axis

POA dysfunction induces hypogonadal osteoporosis. Congenitally gonadotropin-releasing hormone (GnRH)-deficient (*hpg*) mice exhibit both hypogonadism and reduced BMD. However, transplantation of POA tissue containing GnRH-positive neurons into the third ventricle of *hpg* mice fully restores the HPG axis, reversing gonadal dysfunction and completely normalizing BMD to wild-type (WT) levels. This demonstrates that the POA promotes bone formation by regulating GnRH secretion (Rajendren et al., 2006).

##### 2.1.6.2 The ERα regulates tamoxifen-induced disruption of bone metabolism

In breast cancer models, tamoxifen acts on POA ERα to trigger gene expression reprogramming, which directly drives bone loss. Conversely, conditional ERα ablation in the POA completely blocks the skeletal effects of tamoxifen, offering a novel strategy to mitigate or avoid this adverse drug effect (Table 1) (Zhang et al., 2021).

**Table 1:** The hypothalamic nuclei regulate bone metabolism.

Nuclei	Subregion or neuron	Intervention or regulatory pathway	Effect on bone	References
ARC	AgRP^+^ neuron	*PDK1* global knockout (*PDK1^−/−^*) mice	Reduce bone mass	Sasanuma et al. (2017)
		*UCP2* global knockout (*UCP2^−/−^*) mice	Reduce bone mass	Kim et al. (2015)
		*Sirt1* conditional knockout in AgRP^+^ neuron	Reduce bone mass	Kim et al. (2015)
		Ablation of *AgRP*^+^ neuron	Reduce bone mass	Kim et al. (2015)
		Deletion of AgRP gene	Promote bone formation	Enriquez et al. (2021)
	NPY^+^ neuron	*NPY* global knockout (*NPY^−/−^*) mice	Promote bone formation	Meunier et al. (2009)
	AgRP^+^/NPY^+^ neuron	ΔFosB antagonizes AgRP^+^/NPY^+^ neuron	Enhance energy expenditure and promote bone formation	Idelevich et al. (2018)
	POMC^+^ neuron	*ERα* conditional knockout in POMC^+^ neuron	Promote bone formation	Ohlsson et al. (2016)
	CART^+^ neuron	*Cart* global knockout (*Cart^−/−^*) mice	Increase bone resorption	Elefteriou et al. (2005)
	POMC^+^/CART^+^ neuron	ΔFosB antagonizes POMC^+^/CART^+^ neuron	Enhance energy expenditure and promote bone formation	Idelevich et al. (2018)
	Kiss1^+^ neuron	*ERα* conditional knockout in Kiss1^+^ neuron	Promote bone formation in intact and OVX female mice	Herber et al. (2019)
		CCN3 upregulates during lactation	Promote bone formation	Babey et al. (2024)
	MSG^+^ neuron	Ablation of MSG^+^ neuron	Induce hypogonadism but does not reduce bone mass	Elefteriou et al. (2003)
VMH	SCTR	SCT inhibits the SNS	Promote bone formation	Zhang et al. (2024)
	HTR2C	BDS inhibits the SNS	Promote bone formation	Oury et al. (2010)
	VMHdm	The VMHdm signal stimulates the SNS	Induce chronic stress-induced bone loss	Yang F. et al. (2020)
	IFT88	Deletion of *IFT88* gene inhibits the SNS	Promote bone formation	Sun et al. (2021)
	TH^+^ neuron	Orthodontic force stimulates the SNS	Accelerate alveolar bone remodeling	Cao et al. (2020)
	SF1^+^ neuron	Leptin stimulates the SNS	Inhibit bone formation	Takeda et al. (2002)
		ΔFosB antagonizes SF1^+^ neuron	Enhance energy expenditure and reduce bone mass	Idelevich et al. (2018)
	Astrocyte	Gq pathway activates SF1^+^ neuron	Increase anxiety-like behaviors and reduce bone mass	Liu et al. (2021)
	VMH integrity	Ablation of the VMH	Leptin cannot reduce bone mass by *i.c.v.*	Takeda et al. (2002)
			Counteract unloading-induced bone loss	Hino et al. (2006)
SCN	Circadian rhythm	Continuous light exposure in mice	Induce early osteoporotic alterations	Lucassen et al. (2016)
		Circadian misalignment in shift workers	Reduce BMD and elevate fracture risk	Quevedo and Zuniga (2010), Feskanich et al. (2008)
	Per2	*Per2* global knockout (*Per2^−/−^*) mice	Promote bone formation	Abe et al. (2019), Fu et al. (2005)
	Y6R	*Y6R* global knockout (*Y6R^−/−^*) mice	Reduce bone mass	Khor et al. (2016)
PVN	OT	OT upregulates BMP2	Enhance osteogenic capacity	Tamma et al. (2009)
	AVP	AVP suppresses osteogenic gene expression	Weaken osteogenic capacity	Sun et al. (2015)
	Avpr1α	*Avpr1α* global knockout (*Avpr1α*^−/−^) mice	Promote bone formation	Sun et al. (2015)
	TH^+^ neuron	Skeletal interoception inhibits the SNS	Promote bone formation	Gao et al. (2024)
LH	OX2R	Orexin reduces circulating leptin levels	Promote bone formation	Wei et al. (2014)
	OX1R	Orexin suppresses local ghrelin levels	Inhibit bone formation	Wei et al. (2014)
		Orexin activates OX1R-Nrf2/HIF-1α pathway	Reverse bone loss induced by chronic intermittent hypoxia	Gu et al. (2022)
	MCH^+^ neuron	MCH^+^ neuron stimulates BMSCs	Promote bone formation	Guo et al. (2024)
POA	GnRH^+^ neuron	Transplant POA into *GnRH* global knockout (*hpg*) mice	Reverse gonadal dysfunction and bone loss	Rajendren et al. (2006)
	ERα	Tamoxifen acts on ERα	Reduce bone mass	Zhang et al. (2021)

### 2.2 Other nuclei of diencephalon

#### 2.2.1 Subfornical organ (SFO)

The SFO is a CVO situated at the midline of the third ventricle floor, dorsal to the anterior commissure. Located outside the BBB, the SFO is one of the few sites in the CNS that directly interfaces with circulating substances, playing a vital role in body fluid homeostasis (Rossi et al., 2019; Mimee et al., 2013; Dai et al., 2013). By sensing systemic sodium ion concentration, the SFO precisely regulates water-electrolyte balance. It contains two functionally antagonistic neuronal populations: activation of GABAergic neurons significantly suppresses drinking behavior, conversely, activation of glutamatergic neurons triggers it (Hiyama and Noda, 2016; Oka et al., 2015). Interestingly, the reciprocal activity between these populations regulates bone homeostasis. Activation of GABAergic neurons suppresses PTH secretion and reduces bone mass; conversely, activation of glutamatergic neurons significantly elevates PTH levels and increases bone mass. The core mechanism involves projections from SFO GABAergic neurons to the PVN, forming a central inhibitory pathway that subsequently modulates PTH secretion and bone metabolism (Zhang et al., 2023).

#### 2.2.2 Pineal gland (PG)

The PG, a neuroendocrine organ situated near the geometric center of the human brain (Reiter, 1993), serves as the primary site of melatonin synthesis in vertebrates and regulates bone metabolism through the secretion of this hormone. Melatonin biosynthesis begins with hydroxylation catalyzed by tryptophan hydroxylase 1 (TPH1), followed by a series of enzymatic cascade reactions. The synthesized melatonin mediates its diverse physiological functions by activating the melatonin receptor 1A (MT1) and 1B (MT2) (Borjigin et al., 2012; von Gall et al., 2002).

##### 2.2.2.1 The PG primarily regulates bone metabolism through the MT2 pathway

*MT2*-specific knockout mice exhibit significantly reduced bone mass, a phenotype absent in MT1-deficient models, definitively revealing the specific role of MT2 in bone homeostasis. This regulatory mechanism is conserved across species: mice with PG-specific *Tph1* deficiency display a sharp decline in bone mass due to decreased melatonin levels (Sharan et al., 2017), a finding consistent with the bone loss observed in pinealectomized sheep models (Egermann et al., 2011).

##### 2.2.2.2 Melatonin deficiency is a potential risk factor for bone loss

Clinically, age-related decline in melatonin secretion is associated with osteoporosis in observational studies (Li et al., 2019). For instance, PMW exhibit lower nocturnal serum melatonin concentrations than perimenopausal women, and the duration of melatonin secretion is often shorter after menopause (Toffol et al., 2014). Given these mechanistic and clinical insights, dietary melatonin supplementation represents a promising therapeutic strategy for mitigating age-related and postmenopausal osteoporosis (Patel et al., 2021).

## 3 Brainstem

### 3.1 Locus coeruleus (LC)

The LC is a small nucleus located in the dorsolateral pons, deep within the brainstem (Poe et al., 2020). It supplies the neurotransmitter NE via an extensive projection network to areas including the neocortex, HC, and spinal cord, playing a key role in regulating arousal, autonomic function, and neuroinflammation (Benarroch, 2018; Beardmore et al., 2021; Giorgi et al., 2020). The LC primarily regulates bone metabolism by modulating sympathetic tone. Within LC neurons, the transcription factor Forkhead box O1 (FoxO1) is highly expressed and drives catecholamine synthesis, particularly NE. Studies show that specific knockout of *FoxO1* in Dbh-expressing LC neurons (*FoxO1_Dbh−/−_*) reduces energy expenditure and markedly increases bone mass (Issidorides et al., 2004; Kajimura et al., 2014). Likewise, adiponectin secreted by adipose tissue modulates LC neuronal activity via the FoxO1 pathway. By reducing sympathetic tone, it consequently increases bone mass and decreases energy expenditure (Kajimura et al., 2013; Polito et al., 2020) Furthermore, phosphodiesterase 5A (PDE5A), a key enzyme targeted for treating erectile dysfunction, is highly expressed in sympathetic neurons of the LC. These neurons establish direct projections to the skeletal system to regulate bone mass. PDE5A inhibitors not only promote osteoblast formation peripherally but also centrally suppress LC-mediated sympathetic tone, thereby increasing bone mass (Kim et al., 2020).

### 3.2 Raphe nuclei (RN)

The RN are a group of nuclei distributed near the midline of the brainstem, whose neuronal clusters extend along its entire rostrocaudal axis, spanning from the midbrain and pons to the medulla oblongata (Hornung, 2003). As the primary cluster of central 5-hydroxytryptamine (5-HT) neurons (Cheng et al., 2022), the RN regulate bone metabolism through the 5-HT pathway. The RN synthesize 5-HT via tryptophan hydroxylase 2 (TPH2). *TPH2* knockout (*Tph2^−/−^*) mice exhibit a significant low-bone-mass phenotype, confirming that brain-derived 5-HT is a positive and powerful regulator of bone remodeling (Yadav et al., 2009; Jones et al., 2020). Furthermore, disrupted 5-HT pathway may underlie bone metabolic disorders comorbid with neurodegenerative diseases. Consistent with this, in the human microtubule associated protein tau (*htau*) gene knock-in AD model, overall brainstem TPH levels are downregulated, accompanied by a significant decrease in BMD (Dengler-Crish et al., 2016).

### 3.3 Nucleus tractus solitarius (NTS)

The NTS, located in the dorsal medulla oblongata, comprises a group of sensory nuclei and functions as a key regulator of autonomic signals (Forstenpointner et al., 2022). The NTS forms a critical component of the BNST-VMH-NTS neural circuit. Specifically, SF1 neurons in the VMH project to vesicular glutamate transporter 2 (Vglut2) neurons in the NTS, a connection that ultimately promotes bone loss through the enhancement of SNS activity (Yang F. et al., 2020).

### 3.4 Amygdala (AMY)

The AMY, composed of interconnected subnuclei within the temporal lobe (Brockett et al., 2021), serves as a core limbic structure and a high-order integration center for emotional processing (Ocklenburg et al., 2022). It coordinates the dynamic equilibrium among emotional states, stress responses, mechanical environment, and bone homeostasis through specific subregions.

#### 3.4.1 The AMY mediates emotional modulation of bone mass via NPY

NPY, which is enriched in GABAergic neurons of the AMY, modulates emotional responses and influences bone mass via its receptors (Wood et al., 2016). Specifically, the neuropeptide Y1 receptor mediates anxiolytic effects, whereas the Y2 receptor (Y2R) promotes anxiety (Tasan et al., 2010). Research has found that specific knockout of *Y2R* in the AMY increases bone mass in mice. Intervention with a brain penetrant Y2R small molecule antagonist in OVX mice effectively restored bone mass (Seldeen et al., 2018).

#### 3.4.2 Stress-induced structural plasticity of the AMY affects bone metabolism

Chronic stress induces lasting dendritic hypertrophy in neurons of the basolateral amygdala (BLA), whereas acute stress leads to their dendritic retraction (Zhang et al., 2019). Clinical ^18^F-fluorodeoxyglucose positron emission tomography/computed tomography (^18^F-FDG PET/CT) analysis confirm that the metabolic activity of the AMY is significantly elevated in PMW. This increase shows a positive correlation with psychological stress scale scores (Psychosocial Well-being Index-Short Form, PWI-SF), thereby providing neurobiological evidence for the role of psychological stress in PMW osteoporosis (Pahk et al., 2021).

#### 3.4.3 The AMY integrates mechanical signals to maintain bone homeostasis

The BLA and the medial part of the central nucleus (CeM) possess anatomical connections with the musculoskeletal system, translating mechanical signals into nuclear instructions for bone homeostasis. Typical mechanical unloading events, such as muscle paralysis, decrease serum levels of semaphorin 3A (Sema3A) and the density of sensory nerve fibers, thereby driving bone loss. Chemogenetic activation of BLA neurons can reverse these alterations and effectively mitigate bone loss. While CeM neurons may complement BLA in processing mechanical signals, their specific mechanisms require further investigation (Liu et al., 2022).

### 3.5 Hippocampus (HC)

The HC is located within the hippocampal sulcus, lying immediately inferior to the floor of the temporal horn of the lateral ventricle and superior to the parahippocampal gyrus. It is anatomically divided into head, body, and tail segments and plays a critical role in learning and memory formation (Alves et al., 2022; Olafsdottir et al., 2018; Lisman et al., 2017). While hippocampal high degree of plasticity underlies cognitive function, it also increases vulnerability to injury and stress (Bartsch and Wulff, 2015). Intriguingly, following traumatic brain injury (TBI), hippocampal neurons in CA1 and dentate gyrus (DG) subregions drive osteogenesis by releasing small extracellular vesicles (sEVs) into the circulation. These sEVs are enriched with osteogenic microRNAs, such as miR-328a-3p and miR-150-5p, and are transported to bone to stimulate osteoprogenitor cells, thereby enhancing bone formation and remodeling. Translating this mechanism, a hydrogel delivering sEVs containing miR-328a-3p was developed and shown to significantly accelerate bone repair in a rat bone defect model, highlighting a novel therapeutic strategy for bone regeneration (Xia et al., 2021).

## 4 Basal ganglia

### 4.1 Striatum (STR)

The STR, located in the deep subcortical region of the cerebral hemispheres, is the largest subcortical structure in mammalian brain (Kuzucu et al., 2023; Lanciego et al., 2012). It comprises two distinct functional domains: the dorsal STR (comprising the caudate nucleus and putamen) integrates corticothalamic input to regulate motor programming and cognitive function, whereas the ventral STR (including the nucleus accumben and olfactory tubercle) mediates mesolimbic dopaminergic reward circuits to drive motivated emotional behaviors (Peak et al., 2019; Gonzalez et al., 2023). The role of STR in bone regulation is prominently illustrated by Huntington’s disease (HD), a condition characterized by severe atrophy of the dorsal STR (Yang X. et al., 2020). Clinical studies demonstrate that striatal volume is significantly reduced in premanifest HD individuals, even before overt changes in body composition (BC), and BMD level shows a significant positive correlation with the volume of the right putamen (Turner et al., 2020). Moreover, the reduction in BMD observed in HD patients is independent of conventional BC parameters—such as body weight (BW), height (H), body mass index (BMI), lean body mass (LBM), and fat mass (FM)—suggesting that BMD may serve as a potential biomarker for HD progression (Costa de Miranda et al., 2019). These findings provide novel clues for understanding the systemic manifestations of HD and point to new directions for clinical intervention in skeletal health (Table 2).

**Table 2:** Beyond the hypothalamus, central nuclei in other regions regulate bone metabolism.

Nuclei	Subregion or neuron	Intervention or regulatory pathway	Effect on bone	References
SFO	GABA^+^ neuron	GABA^+^ neuron suppresses PTH secretion	Reduce bone mass	Zhang et al. (2023)
	Glu^+^ neuron	Glu^+^ neuron elevates PTH levels	Promote bone formation	Zhang et al. (2023)
PG	MT2	*MT2* global knockout (*MT2^−/−^*) mice	Reduce bone mass	Sharan et al. (2017)
	TPH1	*Tph1* conditional knockout in PG	Reduce bone mass	Sharan et al. (2017)
LC	Dbh^+^ neuron	*FoxO1* conditional knockout in Dbh^+^ neuron	Decrease energy expenditure and promote bone formation	Kajimura et al. (2014)
	PDE5A^+^ neuron	PDE5A inhibitor reduces sympathetic tone	Promote bone formation	Kim et al. (2020)
RN	TPH2	*Tph2* global knockout (*Tph2^−/−^*) mice	Reduce bone mass	Yadav et al. (2009)
	TPH	*Htau* gene knock-in AD model	Reduce bone mass	Dengler-Crish et al. (2016)
BNST	SOM^+^ neuron	Involve in the BNST-VMH-NTS neural circuit	Induce chronic stress–induced bone loss	Yang F. et al. (2020)
NTS	Vglut2^+^ neuron	Involve in the BNST-VMH-NTS neural circuit	Induce chronic stress–induced bone loss	Yang F. et al. (2020)
AMY	BLA	Activation of BLA neuron	Rescue bone loss induced by mechanical unloading	Liu et al. (2022)
	Y2R	*Y2R* conditional knockout in AMY	Promote bone formation	Seldeen et al. (2018)
HC	CA1 and DG subregions	Release sEVs enriched with miRNAs to target osteoprogenitor cells	Promote bone formation	Xia et al. (2021)
STR	Volume of the striatum	In asymptomatic HD patients	BMD correlates with right putamen volume	Costa de Miranda et al. (2019)

## 5 Brain regional structure

Growing evidence from neuroimaging and genetics suggests that structural alterations in the CNS are associated with bone metabolism. These associations can be categorized into three complementary lines of evidence.

### 5.1 Gray matter (GM) volume correlates with BMD with region and sex specificity

Mendelian randomization (MR) analysis indicates a positive association between the GM volume of the left inferior frontal gyrus (IFG) and pars opercularis (POP) with BMD at the total body, femoral neck, and heel (Guo et al., 2023). Observational studies further reveal that women with lower femoral neck BMD exhibit reduced GM volume in widespread regions, including the superior frontal gyri, middle temporal gyri, HC, and temporal poles. In contrast, the GM volume reduction in men is more limited, primarily involving the subcallosal area, left basal forebrain and entorhinal area (Kalc et al., 2023). Notably, this relationship emerges early in neurodegenerative processes, as seen in the early stage of AD, where lower BMD is linked to reduced GM volume in the hypothalamus and limbic system (Loskutova et al., 2010).

### 5.2 The integrity of white matter (WM) is associated with bone metabolism

Cohort studies have indicated that higher femoral neck BMD is associated with less WM hyperintensity burden, suggesting that WM integrity may contribute to the maintenance of bone homeostasis (Stefanidou et al., 2021).

### 5.3 Genetic studies provide clues to a potential bidirectional link between the brain and bone

MR analysis suggests an association between osteoporosis and alterations in brain cortical structure, including reduced thickness in the precentral region and decreased total cortical surface area (SA). Meanwhile, osteoporosis causally reduces the SA of the global cerebral cortex and the paracentral lobule. Furthermore, there may be potential causal relationships between decreased femoral neck BMD and reduced total cortical SA, as well as between vulnerability to osteoporosis and reduced thickness in the Parstriangularis region (Nie et al., 2024; Li L.-J. et al., 2024).

In summary, the above evidences strongly support the link between structural changes in the CNS and bone metabolism. However, current clues primarily stem from observational studies and genetic inferences. The exact causal mechanisms and specific neurobiological pathways still require further systematic basic research and rigorously designed clinical studies to be elucidated.

## 6 Nuclear regulatory network

Previous sections have elaborated that central nuclei distributed across broad brain regions, including the diencephalon, brainstem, limbic system, and basal ganglia, exert precise regulation of bone metabolism through neural or endocrine pathways. However, maintaining bone homeostasis is not solely dependent on the independent function of any single nucleus. In fact, these functionally diverse and spatially distributed nuclei form a highly interconnected network. By establishing neural circuits or sharing signaling molecules, they integrate information pertaining to the energy status, circadian rhythms, mechanical loading, emotional stress, and fluid balance. Ultimately, these interactions converge into coherent nuclear commands that precisely regulate the balance between bone formation and bone resorption. A deeper understanding of the modes and mechanisms of nuclei communication is essential for deciphering the overall regulatory logic of the BBA and for elucidating the pathogenesis of bone metabolic disorders. This section will analyze several representative modes of nuclear interaction to illustrate how they collaboratively regulate bone metabolism.

### 6.1 Leptin maintains bone homeostasis by coordinating antagonistic nuclei

Leptin secreted by adipocytes activates VMH neurons, which inhibit osteoblast proliferation via the SNS. Leptin can also activate ARC neurons to suppress bone resorption by increasing CART expression (Karsenty, 2006). Additionally, leptin acts on RN to inhibit bone formation by reducing the synthesis of BDS.

The weight of these parallel pathways is not fixed, but is highly dependent on the energy status. Under conditions of leptin deficiency (e.g., in *ob/ob* mice) or leptin resistance, despite a tendency toward enhanced osteogenic activity (Ducy et al., 2000), the expression of NPY in the ARC is markedly elevated. NPY exerts a potent anti-osteogenic effect via Y2 receptor and counteracts the inhibitory signals induced by leptin (Baldock et al., 2002). This reveals a sustained crosstalk between leptin and other central nuclei, which collectively determine the output of bone metabolism.

CART represents a critical compensatory node within this network. When other components (e.g., the MC4R) are functionally deficient, the expression of CART in the ARC is upregulated in a compensatory manner, which has been identified as a backup for preserving bone homeostasis and preventing bone loss (Ahn et al., 2006). This clearly demonstrates how the leptin-mediated network achieves functional compensation by mobilizing components within its own molecular repertoire.

**Figure 4:**
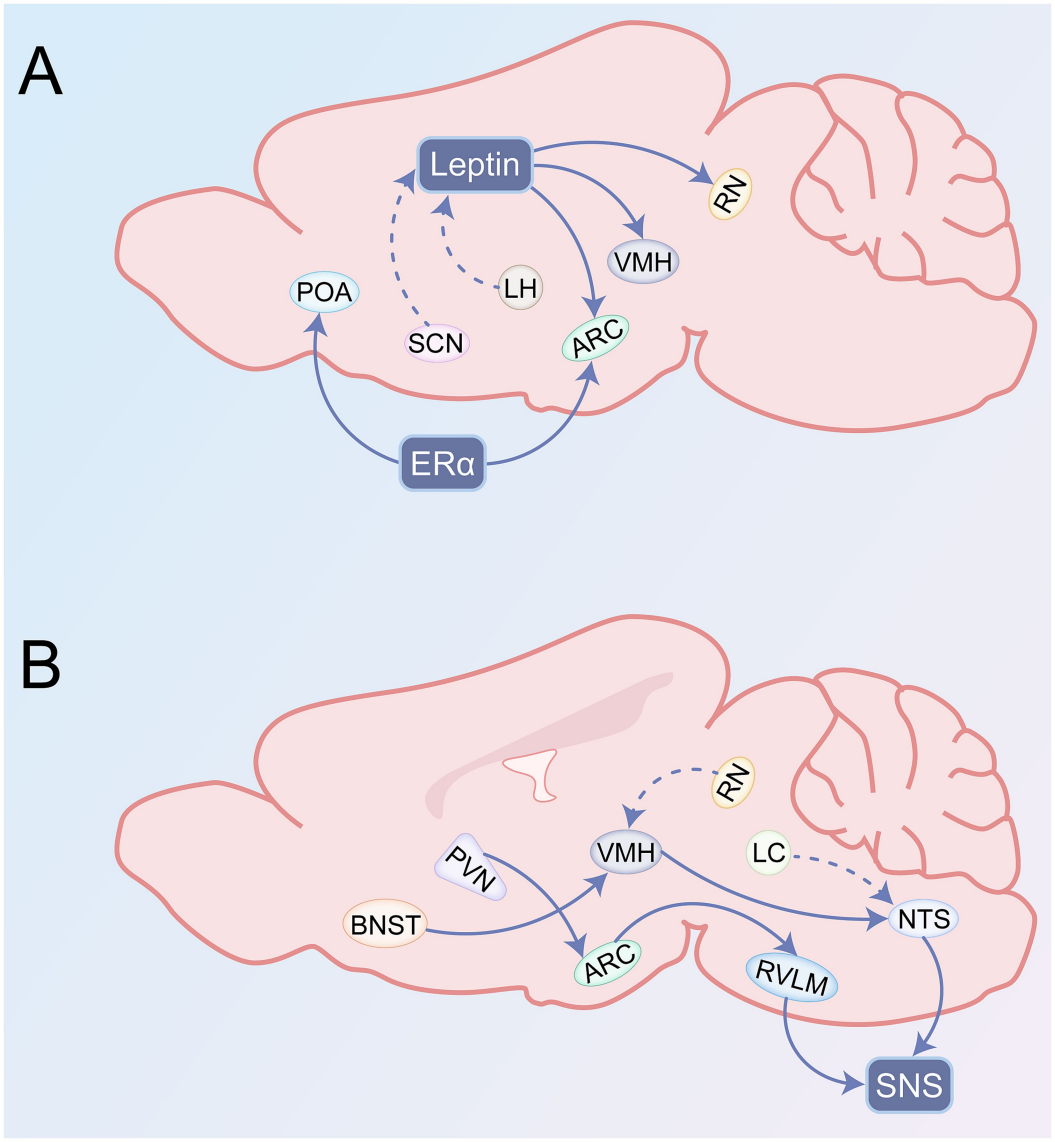
**(A)** Leptin- and ERα-mediated nuclear network of bone metabolism. Leptin activates the VMH to inhibit osteoblast proliferation, while stimulating the ARC to suppress bone resorption. It acts on the RN to reduce bone formation. The SCN maintains circadian leptin fluctuations, while the LH can reduce leptin levels. ERα activation in the ARC inhibits bone mass. Furthermore, ERα in the POA mediates bone loss. **(B)** SNS-mediated nuclear network of bone metabolism. The PVN integrates inputs from the ARC to descend projections that relay through the RVLM, while the VMH conveys inputs from the BNST to the NTS. These signals ultimately activate the SNS to reduce bone mass. The LC and RN function as effector amplifiers and buffer stabilizers, respectively, modulating SNS-mediated bone remodeling.

Molecular switches within this network precisely regulate the direction and intensity of signal transmission. Leptin secretion itself exhibits circadian rhythmicity modulated by the SCN, and clock gene is a prerequisite for leptin-mediated inhibition of bone formation through the SNS (Kalsbeek et al., 2001). Furthermore, orexin secreted by LH neurons reduces circulating leptin levels via central pathways, thereby indirectly attenuating the suppression of bone formation mediated by the VMH neurons (Wei et al., 2014) (Figure 4A).

### 6.2 The SNS regulates bone metabolism hierarchically via multiple nuclei

The PVN acts as a high-level command center for sympathetic activity, modulating its output via descending fibers from the pre-autonomic neurons. These projections either indirectly relay through the RVLM or directly target SPNs in the spinal cord, thereby fine-tuning sympathetic tone. Upon receiving mechanical loading signals from the ARC, the PVN significantly suppresses TH expression, leading to reduced sympathetic activity and enhanced bone formation. Acting as a signal transducer, the VMH conveys GABAergic inputs from the BNST to the NTS, which ultimately activates the SNS to inhibit bone formation. The LC functions as an effector amplifier by upregulating Dbh expression and dramatically boosting NE synthesis efficiency, thereby amplifying the effects of upstream signals on bone metabolism.

Under chronic stress conditions, the weight of a circuit involving the central nucleus of the amygdala (CeA) increases drastically. Studies have demonstrated that chronic social defeat stress specifically activates corticotropin-releasing hormone (CRH)-expressing neurons in the CeA, which in turn hyperactivate NE neurons in the LC. This pathway emerges as the dominant circuit driving sympathetic output and consequent bone loss (Zhao et al., 2025). This indicates that under pathological conditions, limbic nuclei (e.g., the CeA) can override the conventional network (e.g., the PVN and ARC) and act as the upstream commander governing SNS-mediated effects of bone mass.

When bone loss pathways within this network are overactivated, the system initiates compensatory mechanisms to preserve bone homeostasis, in which the RN plays a central role. The RN releases 5-HT to downstream nuclei (e.g., the VMH), activating signaling cascades via HTR2C to directly suppress SNS hyperactivity (Oury et al., 2010). The activity of the RN provides a critical buffer against excessive sympathetic activation.

The dynamics of this network are governed by key molecular switches. The β_2_-ARs on osteoblasts and osteoclasts represent the primary switch that transduces sympathetic NE signals into cellular responses (Elefteriou, 2018). TH, the rate-limiting enzyme in NE synthesis, acts as an upstream switch controlling the intensity of sympathetic signals broadcast to the skeleton (Kuhn et al., 2022) (Figure 4B).

### 6.3 ERα suppresses bone formation via negative feedback within specific nuclei

ERα primarily promotes bone formation and inhibits bone resorption at the peripheral level. However, specific deletion of ERα within POMC neurons of the ARC leads to increased bone mass in mice. Meanwhile, ERα within ARC Kiss1 neurons also exerts an inhibitory effect on bone formation. Furthermore, ERα in the POA mediates tamoxifen-induced bone loss, and its specific knockout blocks this effect.

The activity of this network undergoes marked changes across developmental stages. During puberty onset, the activity of neurons Kiss1 and neurokinin B in the ARC is enhanced to drive the reproductive axis (Myers et al., 2016). Notably, approximately 95% of Kiss1 neurons co-express ERα, and this pattern is established during early postnatal life (Watanabe et al., 2024). Thus, the elevated estrogen levels during puberty may, via the activation of ERα, reinforce the signals of bone metabolism while promoting reproductive maturation.

When the function in this network is compromised, other nuclei may exert compensatory effects. ERα in the POA is indispensable for generating the luteinizing hormone surge and maintaining the estrous cycle (Watanabe et al., 2024). In contrast, ERα in the ARC primarily mediates the inhibition of estrogen on the GnRH pulse generator (McQuillan et al., 2022). These results indicate that the spatial functional partitioning of ERα confers compensatory potential for the regulation of bone metabolism.

Multiple tiers of molecular switches precisely govern this network. Neurons in the ARC and the POA exhibit differential sensitivity to estrogen. ARC neurons respond to low doses of estrogen that are sufficient to suppress LH pulses, whereas POA neurons require high estrogen doses to induce positive feedback and the luteinizing hormone surge (Henriques et al., 2022). This implies that fluctuations in circulating estrogen levels switch the output state of the entire network, thereby modulating bone metabolism (Figure 4A).

## 7 Conclusions and future perspectives

This review systematically elucidates the core mechanisms of the BBA, revealing the decisive role of central nuclei in regulating bone metabolism. First, it transcends the conventional “hypothalamo-centric” perspective by revealing how central nuclei distributed across various brain regions modulate bone remodeling through diverse pathways. Next, it synthesizes key translational evidence strongly supporting the clinical relevance of central nuclei in regulating bone metabolism. Finally, it synthesizes the crosstalk and communication between central nuclei, demonstrating that their collaboration forms a dynamic regulatory network essential for maintaining bone homeostasis.

Advances in science and technology have laid a solid foundation for unraveling the precise mechanisms by which central nuclei remotely regulate bone metabolism, profoundly driving the evolution of research paradigms in the BBA. Techniques such as optogenetics, chemogenetics, neural circuit tracing, and gene editing have enabled spatiotemporally precise manipulation of specific nuclei, confirming their pivotal role in bone remodeling. Meanwhile, the integrated application of cutting-edge technologies—including high-resolution computed tomography (HRCT) (Burghardt et al., 2011), *in vivo* calcium imaging (Resendez and Stuber, 2015), and spatial transcriptomics (Jung and Kim, 2023)—has progressively unraveled the complete pathway through which nuclear commands are transmitted to the skeleton. These technological breakthroughs not only provide strong theoretical support for the BBA but also propel scientific research from phenomenal observation into mechanistic decoding. However, critical research gaps remain in this field. First, the process underlying the transition of central adaptive regulation of bone metabolism to pathological dysregulation remains elusive. In cases of chronic stress, circadian rhythm disruption, AD and postmenopausal osteoporosis, the adaptive regulation of bone metabolism gradually progresses to irreversible pathological impairment due to the dysfunction of corresponding central nuclei, the failure of neural pathway regulation and the dysregulation of receptor-mediated negative feedback mechanisms. Meanwhile, there is still a lack of quantifiable multi-dimensional biomarkers for defining the threshold of bone metabolic decompensation, which encompasses key dimensions such as the structural plasticity of central nuclei, sympathetic neural activity and intrinsic skeletal repair capacity. Among these, dendritic hypertrophy in the BLA and degeneration of clock neurons in the SCN represent typical manifestations of abnormal structural plasticity of central nuclei, whereas the sustained excessive synthesis of NE constitutes the feature of abnormal sympathetic neural activity. Furthermore, core molecular switches serve as the key basis for the regulation of bone metabolism by central nuclei, and the regulatory mechanisms underlying their functional abnormalities remain poorly elucidated. Such molecular abnormalities manifest as the dysregulated phosphorylation of CREB, the suppressed expression of FoxO1, the disrupted rhythm of Per2 and the inactivation of ERα, which also act as important molecular inducers driving the transition of bone metabolism to pathological dysregulation. Finally, the regulatory basis for individual differences in this pathological transition process of bone metabolism remains undefined. Genetic background, sex hormone levels, age-related functional decline and underlying comorbidities have all been identified as potential driving factors, which directly hampers the translational application of basic research findings into personalized clinical interventions (Liu et al., 2025).

Besides, elevating the significance of this field, nuclei-targeted modulation has shown encouraging preclinical efficacy. Optogenetics and chemogenetics realize spatiotemporally precise nuclear regulation, effectively reversing chronic stress-related bone loss (Zhao et al., 2025). Several other approaches also exhibit great therapeutic potential. Deep brain stimulation (DBS) has already demonstrated therapeutic efficacy in the treatment of patients with Parkinson’s disease (Hariz and Blomstedt, 2022), and it may be applied to the regulation of bone metabolism by targeting deep brain nuclei such as the hypothalamus in the future. Nuclei-targeted drug delivery systems (nanoparticles, exosomes) address the poor targeting and low BBB crossing efficiency of conventional therapies (Ma et al., 2025). Non-invasive neuromodulation (e.g., transcranial magnetic stimulation, vagus nerve stimulation) is highly suitable for clinical translation owing to its safety and good patient compliance (Guo et al., 2023), indirectly regulating central circuits to improve bone metabolism. Despite promising preclinical outcomes, nucleus-targeted therapies confront four major translational bottlenecks. First, precise targeting of deep, small, anatomically indistinct nuclei is quite difficult, carrying risks of off-target effects and metabolic disorders. Second, most preclinical studies adopt young, genetically homogeneous rodents, while clinical patients are mostly elderly, comorbid and heterogeneous. Third, no standardized biomarkers are available for patient stratification or real-time efficacy monitoring. Fourth, invasive techniques pose safety risks, and long-term neuromodulation may trigger neural circuit adaptation, weakening therapeutic efficacy (Gerosa and Lombardi, 2021).

To address the aforementioned challenges, future research urgently requires a paradigm shift in two core dimensions. At the theoretical level, it is essential to establish a spatiotemporal multi-layered regulatory network of brain-wide central nuclei. This begins with deciphering the hierarchical upstream-downstream regulatory link between core molecular switch dysfunction and neural circuit pathological remodeling, and proceeds to constructing a quantifiable multi-dimensional biomarker system for the decompensation threshold to enable early pathological identification. Ultimately, integrating spatial topological connection maps of central nuclei and temporal dynamics will help elucidate the spatiotemporal correlation between nuclear activity and bone metabolism. At the translational level, there is an urgent need to drive the deep integration of nucleus-targeted modulation with skeleton-targeted therapies, with key breakthroughs to be achieved across complementary research directions. High-precision targeting tools should be developed via neuroimaging and optimized viral vectors to minimize off-target effects, while therapeutic strategies require validation in aged, comorbid animal models alongside the advancement of early clinical trials for refractory osteoporosis. Concurrently, non-invasive, long-acting neuromodulation delivery systems ought to be optimized to balance efficacy and safety. Specifically, bioactive materials such as magnesium-containing scaffolds and piezoelectric composites can mediate neuro-osseous crosstalk to enhance bone regeneration; targeted drug delivery systems loaded with neuropeptides (e.g., SP, CGRP) or neurotrophins (e.g., NGF) enable sustained local release, improving the repair effect of bone defects; cell-based therapies like Schwann cell and BMSC co-culture promote neural innervation and osteogenesis (Li J. et al., 2024). Only by building such a systematic research platform that integrates technological development, mechanistic exploration and clinical translation can BBA research completely break through the constraints of fragmented insights, propel the field from isolated breakthroughs to holistic integration, and formally usher in a new era of precisely decoding the whole-brain central nuclear regulatory networks.

## Glossary

AD: Alzheimer’s disease
AgRP: Agouti-related peptide
AMY: Amygdala
AP1: Activator protein 1
ARC: Arcuate nucleus
BBA: Brain-Bone Axis
BBB: Blood–brain barrier
BDS: Brain-derived serotonin
BLA: Basolateral amygdala
BMD: Bone mineral density
BNST: Bed nucleus of the stria terminalis
CART: Cocaine -and amphetamine-regulated transcript
CeM: Medial part of the central nucleus
CNS: Central nervous system
CREB: CAMP response element-binding protein
DG: Dentate gyrus
ER*α*: Estrogen receptor α
FGF23: Fibroblast growth factor 23
FoxO1: Forkhead box O1
GC: Glucocorticoid
GM: Gray matter
GnRH: Gonadotropin-releasing hormone
HC: Hippocampus
HPG: Hypothalamic–pituitary-gonadal
IGF1: Insulin-like growth factor 1
Kiss1: Kisspeptin
LC: Locus coeruleus
LCN2: Lipocalin 2
LH: Lateral hypothalamus
NE: Norepinephrine
NPY: Neuropeptide Y
NTS: Nucleus tractus solitarius
*ob/ob*: Leptin-deficient
OCN: Osteocalcin
OVX: Ovariectomized
*Per*: *Period*
PG: Pineal gland
PMW: Postmenopausal women
POA: Preoptic area
POMC: Pro-opiomelanocortin
PVN: Paraventricular nucleus
rAAV: Recombinant adeno-associated virus
RANKL: Receptor activator of nuclear factor kappa B ligand
RN: Raphe nuclei
RVLM: Rostral ventrolateral medulla
SCN: Suprachiasmatic nucleus
SF1: Steroidogenic factor 1
SFO: Subfornical organ
SNS: Sympathetic nervous system
SON: Supraoptic nucleus
STR: Striatum
TH: Tyrosin hydroxylase
VMH: Ventromedial hypothalamus
VMHdm: Dorsomedial VMH
WM: White matter
WT: Wild-type
β_2_-AR: β_2_-adrenergic receptor
